# Speed of processing training in middle-aged and older breast cancer survivors (SOAR): results of a randomized controlled pilot

**DOI:** 10.1007/s10549-017-4564-2

**Published:** 2017-11-11

**Authors:** Karen Meneses, Rachel Benz, Jennifer R. Bail, Jacqueline B. Vo, Kristen Triebel, Pariya Fazeli, Jennifer Frank, David E. Vance

**Affiliations:** 1 School of Nursing, University of Alabama at Birmingham, Birmingham, AL USA; 2 School of Health Professions, University of Alabama at Birmingham, Birmingham, AL USA; 3 School of Medicine, University of Alabama at Birmingham, Birmingham, AL USA; 4 Comprehensive Cancer Center, University of Alabama at Birmingham, Birmingham, AL USA; 5 Center for Translational Research on Aging and Mobility, University of Alabama at Birmingham, Birmingham, AL USA; 6 Office of Research and Scholarship, School of Nursing, University of Alabama at Birmingham, Medical Towers 501H1, 1720 Second Avenue South, Birmingham, AL 35294-4410 USA

**Keywords:** Cognitive changes, Cognitive impairment, Breast cancer survivors, Aging, Speed of processing interventions

## Abstract

**Purpose:**

Cognitive changes are common among breast cancer survivors. There is limited evidence to guide management of cognitive changes. This randomized controlled pilot evaluated the preliminary efficacy of a speed of processing (SOP) training among middle-aged and older breast cancer survivors.

**Methods:**

Sixty breast cancer survivors  with self-reported cognitive changes were recruited to the SOAR study. Participants were randomized to either a home-based SOP training (*n* = 30) or no-contact control group (*n* = 30). Primary outcomes were SOP (Useful Field of View Test^®^), and executive function (NIH Toolbox Cognition Battery). Neuropsychological assessments were completed at baseline, 6 weeks, and 6 months post study entry. Data were analyzed using repeated measures *t* tests, analysis of covariance, and sensitivity analyses.

**Results:**

SOP training resulted in improvement in objective measures of SOP and executive function. Immediate (6 week) posttest and 6-month follow-up demonstrated large SOP training effects over time. Large representation of African American women (51.2%) and 96% retention in the SOAR study add to study strengths.

**Conclusion:**

Home-based SOP training shows promise for remediating cognitive changes following breast cancer treatment, particularly improved SOP, and executive function.

## Background

Breast cancer is primarily a disease of aging with approximately 66.4% of newly diagnosed cases occurring in women over the age of 55 years [1]. Advances in treatment have led to excellent survival and life expectancy of 20 years or more after completing treatment [2]. Unfortunately, successfully treated aging women with breast cancer are not necessarily living better. Cognitive deficits are one of the more troubling late effects of treatment with incidence ranging from 21 to 90% [3, 4]. Increasing evidence indicates cognitive deficits occur primarily in speed of processing (SOP) as well as in the domains of memory, attention, and executive functioning [5].

Evidence from the cognitive neuroscience literature clearly demonstrates that various types of computerized cognitive training protocols are effective in improving cognitive functioning in a number of cognitive domains among multiple patient populations [6]. In the ACTIVE Study, the largest study of cognitive training in community-dwelling older adults (*N* = 2802), participants were randomized to receive 10 h of training in one of three domains, SOP, executive functioning, or memory [5]. All three groups demonstrated improvement on tasks in the domain of their training. Of the three cognitive training protocols, SOP training had the most robust therapeutic gains and also improved other outcomes such as everyday functioning, driving ability and driving safety, and quality of life indicators. Accumulating data from the ACTIVE Study indicate that cognitive training, specifically SOP training, helps older adults to age better cognitively, even 10 years after receipt of training [7]. Since this intervention works well in older adults [8], such targeted cognitive training may be of value for older breast cancer survivors (BCS) [9, 10].

Breast cancer survivors (BCS) have a high incidence of impairment on neurocognitive tests of SOP [10, 11]. Even subtle SOP deficits can disrupt other cognitive domains such as memory and attention, thus impeding everyday functioning at work and home [11]. Fortunately, a commercial SOP training intervention, performed either at home or in a clinical setting, has demonstrated improvement in the rate at which healthy older adults process information [12]. In an integrative review of 21 cognitive interventions with BCS, Vance and colleagues [9] found only one study used SOP training. In that study, with a predominantly Caucasian sample (*n* = 72; 88%), Von Ah and colleagues [11] observed that 10 h of SOP training was effective in improving speed of processing as well as memory over a 2-month period. Participants completed the SOP training in small groups in a university setting. Building on the findings, the current study investigated whether a home-based, computerized SOP training intervention would improve cognition in a more racially diverse sample of middle-aged and older BCS over a 6-month period. Furthermore, given the geographic location in the Deep South, the authors anticipated a higher percentage of African American BCS participation than is typical in many studies of cognition in BCS.

## Methods

The Speed of Processing in Middle-Aged and Older Breast Cancer Survivors (SOAR) Study was conducted at the University of Alabama at Birmingham (UAB). The research protocol was approved by the UAB Institutional Review Board (IRB).

### Study eligibility

Inclusion criteria consisted of the following: breast cancer diagnosis, ≥ 21 years of age, English speaking, ≥ 6 months post primary breast cancer treatment, and having computer and internet connection access. Exclusion criteria consisted of the following: stage IV metastatic breast cancer, significant neuro-medical comorbidities (e.g., schizophrenia, epilepsy, bipolar disorder, post-traumatic stress syndrome, Alzheimer’s disease or related dementias; AIDS-related dementia; diagnosis of mental handicap; diagnosis of metastatic breast cancer), or conditions that could impact cognitive functioning or testing (e.g., currently enrolled in a residential substance abuse treatment, legally blind or deaf, currently undergoing radiation or chemotherapy, and a history of brain trauma with a loss of consciousness greater than 30 min).

### Procedures

BCS were recruited via several methods including: flyer announcements at the local cancer center outpatient department, community and advocacy events geared towards BCS, and word of mouth. Interested BCS provided their names and contact information to study personnel. Those wishing to participate were first screened for study eligibility using a telephone screening tool. Eligible BCS received a welcome packet and a copy of the informed consent via mail. The welcome packet allowed participants ample time to consider participation and to complete the self-report questionnaires. Next, participants were scheduled for an in-person appointment to give written informed consent and to undergo objective cognitive function testing (i.e., computerized neuropsychological assessment).

All neuropsychological assessments was conducted at the UAB Edward R. Roybal Center for Translational Research on Aging and Mobility and were administered by trained research staff. The neuropsychological assessments included: the Useful Field of View Test and the NIH Toolbox Cognition Battery. Upon completion of the neuropsychological assessments, participants were randomly assigned to either a home-based SOP training group (*n* = 30) or a no-contact control group (*n* = 30). The same neuropsychological assessments was administered immediately post intervention (roughly 6–8 weeks after baseline) and at a 6-month follow-up. All participants were compensated $50 for each of the three data collection visits completed. In addition, intervention participants received $20 for each hour of 10 h of SOP training completed.

### SOAR intervention

Participants accessed the SOP training using their home computer as trained by the research associate. The SOP training used the commercially available “Double Decision” program (www.BrainHq.com) originally developed as part of the ACTIVE Study and then refined over time [12]. This program systematically reduces the stimulus duration during a series of progressively more difficult information-processing tasks presented via computer. During training, participants were evaluated on their level of proficiency (speed and accuracy) on the “Double Decision” task, which involves identifying a central target (either a car or a truck) and noticing where a peripheral target was located in conditions with varying degrees of difficulty (i.e., increased distractors in the periphery or the addition of another central target). The exercises automatically adjust to user performance to maintain a 75% correct rate during the training session in order to promote motivation and a sense of accomplishment for participants.

Participants in the intervention group were instructed to complete 2 h of SOP training per week for a total of 10 h within 6–8 weeks. Participants also received weekly contact via their preferred method (i.e., telephone call, text, or email) to remind them about their SOP training.

#### Sociodemographic and cancer treatment assessment

The investigators used a sociodemographic and cancer treatment questionnaire consisting of 20 items including: age, race, education, marital status, employment status, and family income. Cancer treatment items included type of surgery, chemotherapy, radiation therapy, endocrine therapy, time in survivorship months, surgery type, treatment type, weight gain, and use of support.

### Neurocognitive assessment

#### Useful field of view (UFOV^®^) test

The UFOV^®^ Test is a measure of visual SOP [13] administered via a touch-screen computer and described in detail elsewhere [14]. Briefly, the UFOV^®^ Test consists of four increasingly difficult subtests designed to assess visual SOP under demands of focused attention (subtest 1), divided attention (subtest 2), and selective attention (subtests 3 and 4). Each subtest score denotes the displayed duration, in milliseconds, of the visual stimuli, wherein 75% accuracy was attained. The total score (sum of subtests 1–4) ranges from 68 to 2000 ms, with a lower score indicating a faster SOP.

#### NIH Toolbox Cognition Battery (NIHTB-CB)

The NIHTB-CB is a brief (~ 30 min) comprehensive computerized cognitive assessment, described in detail elsewhere [15]. The battery includes cognitive tests of executive function (Flanker, Dimensional Card Change Sorting), attention (Flanker), episodic memory (Picture Sequence Memory Test), language (Picture Vocabulary Test, Oral Reading Recognition Test), SOP (Pattern Comparison Test), and working memory (List Sorting Test). Given the nature of the intervention, language measures were not examined as outcomes in the current study. The NIHTB-CB generates raw, computed, and uncorrected scaled scores. Consistent with NIHTB-CB recommendations for examining change, this study used raw scores for the List Sorting Test and Pattern Comparison Test and computed scores for all other measures [16].

### Adherence

In line with the ACTIVE Study [6], those who completed ≥ 8 h of cognitive training were considered adherent to SOP training. The online program (www.BrainHQ.com) automatically recorded and stored time and date, performance, and the duration of each training session.

### Statistical analyses

All data were analyzed using SPSS V-23. The significance level was set at 0.05 and was not adjusted for multiple comparisons because this was a pilot study. Preliminary analyses was conducted to examine whether there were any group differences between sociodemographic characteristics, cancer treatment, and survivorship characteristics using *t* tests or Pearson’s Chi square tests when appropriate. T-tests were used to confirm that the two conditions did not differ on baseline performance on the cognitive variables. Repeated measures t-tests were conducted for each cognitive outcome separately within each condition. Confirmatory analysis of covariance (ANCOVA) were conducted controlling for baseline performance for each of the cognitive outcomes to determine whether there was a main effect of condition. Finally, repeated measures analysis of variance (ANOVA) were conducted as sensitivity analyses to the prior analyses to examine the group X time interaction. Cohen’s D effect sizes were calculated on pre-post difference scores (i.e., baseline to 6 week, and baseline to 6 month follow-ups) between the conditions for each cognitive measure; we used the range for Cohen’s D of small effect size as 0.2, medium effect size as 0.5, and large effect size as 0.8 [17].

## Results

### Study sample

**Fig. 1:**
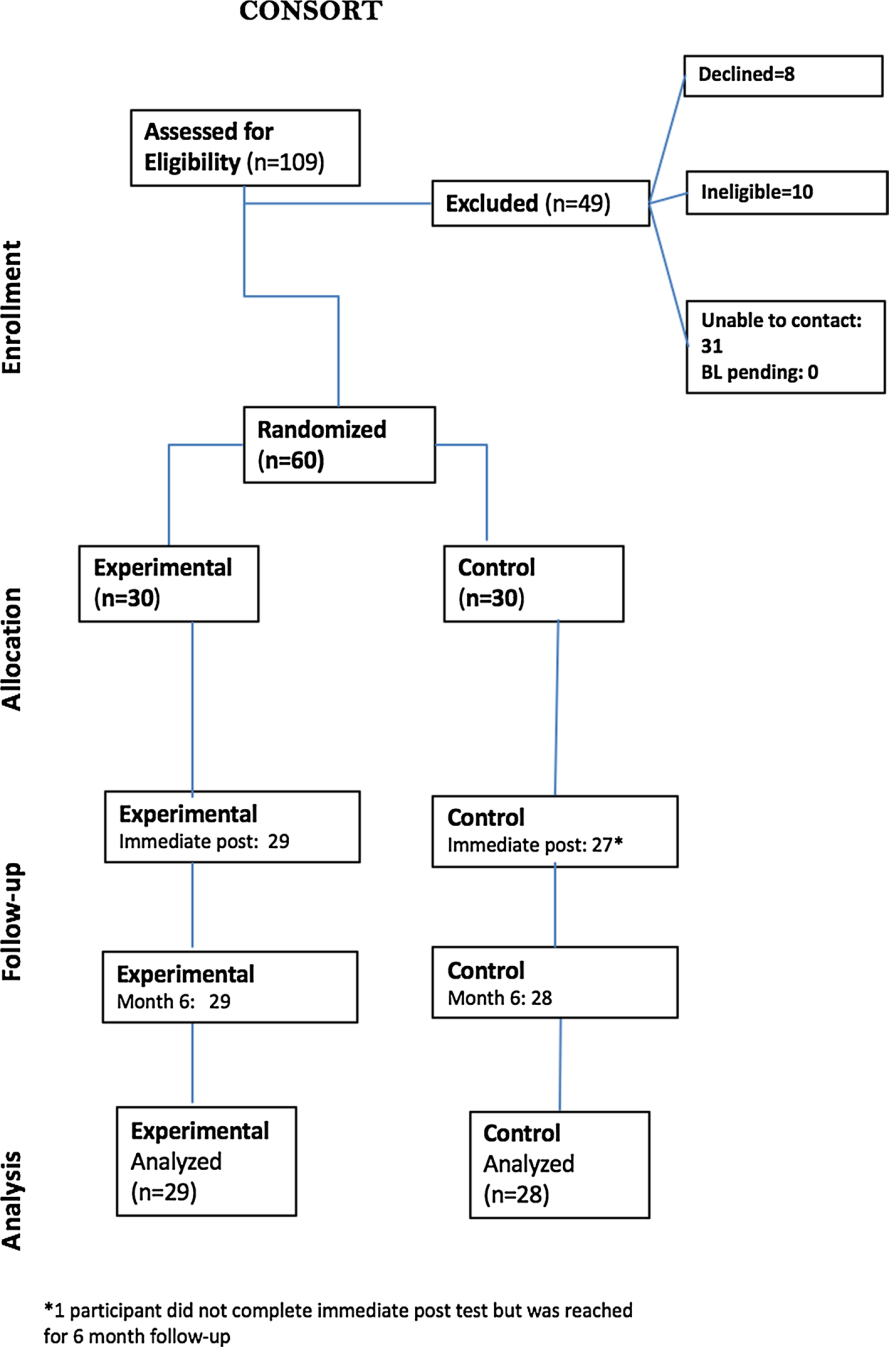
SOAR study consort

Sixty female BCS enrolled between June 2015 and October 2016 (see Fig. 1). All completed baseline assessment. As seen in Table 1, the mean age of the sample was 54.6 years (SD = 10 years). Nearly 52% (*n* = 31) were African American, and 47% were married (*n* = 27). More than 38% (*n* = 23) reported being retired or disabled; and more than 96% (*n* = 58) had health insurance. (See Table 1). Overall, participants in both groups were well-matched on demographic variables with the exception that the SOP training group had significantly more married participants and a significantly greater mean number of cohabitants.

**Table 1:** Sociodemographic Characteristics (*N* = 60)

Variable	Total (*N* = 60)		Control (*n* = 30)		Intervention (*n* = 30)		*p*
	Mean (*SD*) range	*n* (%)	Mean (*SD*) range	*n* (%)	Mean (*SD*) range	*n* (%)	
Age	54.67 (10)		55.65 (9.9)		53.69 (10)		0.45
Race							
African American		31 (51.7%)		15 (50%)		16 (53.3%)	0.80
Caucasian		29 (48.3%)		15 (50%)		14 (46.7%)	
Years of education	15.28 (2.7)		15.47 (2.4)		15.10 (2.9)		0.60
Marital status							
Married		27 (45%)		9 (15%)		18 (60%)	**0.02**
Not married		11 (24.4%)		5 (16.7%)		6 (20%)	
Divorced/widowed		22 (36.6%)		16 (53.3%)		6 (20%)	
Number of cohabitants			1.2 (1.2)		2.1 (1.5)		**0.01**
Employment status							
Employed		31 (51.7%)		17 (56.7%)		17 (56.7%)	0.61
Unemployed		6 (10%)		2 (6.7%)		4 (13.3%)	
Retired/disabled		23 (38.3%)		11 (36.6%)		12 (40%)	
Family income							
<$30,000		14 (23.3%)		7 (23.3%)		7 (23.3%)	0.50
>$30,000		37 (61.7%)		20 (66.7%)		17 (56.7%)	
Do not care to respond		7 (11.7%)		3 (10%)		4 (13.3%)	
Missing		2 (3.3%)		0		2 (6.7%)	
Health insurance							
Insured		58 (96.7%)		29 (96.7%)		29 (96.7%)	1
Not insured		2 (3.3%)		1 (3.3%)		1 (3.3%)	

*p* values in bold text are indicative of significance at *p* < 0.05

As seen in Table 2, regarding survivorship and cancer treatment characteristics, the mean number of years of survivorship was 5.8 years (SD = 5.5 years), and the mean time since completion of cancer treatment was 4.7 years (SD = 5.6 years). More than 73% received support services for cancer treatment. Only one significant difference was found for treatment, with the SOP training group having a higher number of participants undergoing radiation therapy. (See Table 2). Study completion by group was 96% for the SOP training and 93% for the no-contact control.

**Table 2:** Cancer treatment and survivorship characteristics (*N* = 60)

Variable	Total (*N* = 60)		Control (*n* = 30)		Intervention (*n* = 30)		*p*
	Mean (SD)	*n* (%)	Mean (SD)	*n* (%)	Mean (SD)	*n* (%)	
Survivorship years	5.8 (5.5)		6.2 (5.4)		5.3 (5.7)		0.53
Chemotherapy							
Yes		51 (85%)		25 (83.3%)		26 (86.7%)	0.72
No		9 (15%)		5 (16.7%)		4 (13.3%)	
Radiation							
Yes		39 (65%)		15 (50%)		24 (80%)	**0.02**
No		21 (35%)		15 (50%)		6 (20%)	
Anti-hormonal medication							
Yes		28 (46.7%)		14 (46.7%)		18 (60%)	0.30
No		32 (53.3%)		16 (53.3%)		12 (40%)	
Time since treatment completion (years)	4.71 (5.58)		4.9 (5.4)		4.5 (5.8)		0.77
Support services used							
Yes		44 (73.3%)		23 (76.7%)		21 (70%)	0.56
No		16 (26.7%)		7 (23.3%)		9 (30%)	

*p* values in bold text are indicative of significance at *p* < 0.05

### Primary analysis

There were no significant differences between the two groups on any of the cognitive measures at baseline (all *p* values > 0.05). As seen in Table 3, repeated measures t-tests revealed that from baseline to posttest 1, the control group improved on three cognitive outcomes (UFOV^®^ subtests 2 and 4, and the total UFOV^®^ score), while the intervention condition improved on six cognitive outcomes (NIH Toolbox SOP, and executive function, and UFOV^®^ subtests 2, 3, 4, and the total UFOV^®^ score). Analyses for baseline to posttest 2 showed that the control group improved on six measures (NIH Toolbox SOP and episodic memory, and UFOV^®^ subtests 2, 3, 4 and the total UFOV^®^ score). The SOP training group improved on seven measures from baseline to posttest 2 (NIH Toolbox SOP, episodic memory, and executive function, and UFOV^®^ subtests 2, 3, 4 and the total UFOV^®^ score).

**Table 3:** Comparison of outcomes between baseline to 6-week posttest and 6-month posttest using ANCOVA controlling for baseline performance (*N* = 60)

Variable	Control (*n* = 30)			Intervention (*n* = 30)			ANCOVA		Effect size	
	Baseline (*n* = 30)	Posttest 1 (*n* = 27)	Posttest2 (*n* = 28)	Baseline (*n* = 30)	Posttest 1 (*n* = 29)	Posttest2 (*n* = 29)	*p* ^1^	*p* ^2^	*d* ^1^	*d* ^2^
	Mean(SD)			Mean(SD)						
NIH toolbox										
Speed of processing	51 (13.7)	53.8 (13.8)	54.3 (11.7)	51 (14.7)	56.1 (15.9)	56.7 (12.3)	0.52	0.57	0.16	0.06
Working memory	16.3 (2.5)	16.1 (2.7)	16.3 (3.4)	16.4 (2.9)	16.9 (3.4)	16.3 (3.4)	0.31	0.64	0.24	0.15
Episodic memory	461 (92.3)	384.3 (187.9)	491.5 (92.3)	479.2 (101.7)	494.5 (127.9)	521.7 (115.1)	**0.02**	0.56	0.55	0.10
Executive function	8 (0.9)	8.1 (0.9)	8 (0.7)	7.9 (0.8)	8.3 (0.8)	8.3 (0.5)	0.19	**<** **0.01**	0.36	0.55
UFOV										
Subtest 1	33.8 (46.1)	22.2 (15.6)	20.5 (8.3)	30.3 (36.7)	17.6 (1.9)	17.4 (1.3)	0.13	0.06	0.03	0.01
Subtest 2	97.2 (102.1)	58 (62.4)	48.6 (54.5)	87.1 (89.1)	28.2 (25.4)	39.9 (31.6)	**0.02**	0.52	0.18	0.02
Subtest 3	192.7 (110.1)	174.3 (107.7)	157.7 (91)	194 (100.2)	113 (67)	119.3 (78.7)	**<** **0.01**	0.06	0.59	0.36
Subtest 4	349.8 (141.8)	301.1 (140.5)	312.5 (135.8)	349.2 (137)	292.4 (148.7)	250.9 (137.4)	0.76	**0.02**	0.08	0.61
Sum of test 1–4	673.5 (343.1)	555.6 (271.6)	539.3 (245.6)	660.6 (310.6)	451.3 (210.3)	482.4 (236.7)	0.06	**0.03**	0.32	0.38

*p* ^1^ Baseline to posttest 1; *p* ^2^ Baseline to posttest 2

*d* ^1^ Cohen’s D effect size of differences between baseline and posttest 1; *d* ^2^ Cohen’s D effect size of differences between baseline and posttest 2

*p* values in bold text are indicative of significance at *p* < 0.05

ANCOVAs controlling for baseline performance on each of the cognitive outcomes were conducted, with group assignment as our independent variable of interest. Given that there were no group differences on any variables that may have substantially influenced cognitive performance (i.e., race, age, or education) and the small sample size in this pilot study, no additional covariates were entered beyond baseline performance on each of the measures.

ANCOVA results were similar to repeated measures *t* test results. For baseline to posttest 1, main effects were found in NIH Toolbox episodic memory, and UFOV^®^ subtests 2 and 3, with the intervention group demonstrating greater improvement on these measures (except for episodic memory in which the control group actually decreased in performance). From baseline to posttest 2, main effects were found for the intervention group on NIH Toolbox executive function and UFOV^®^ subtest 4 and the UFOV^®^.

Given that results may have been obscured by only examining statistical significance, effect sizes were examined. Our effect size analyses (Cohen’s Ds) revealed from baseline to posttest 1, small to medium effects for NIH Toolbox working memory and executive function; medium to large effects for NIH Toolbox episodic memory and UFOV^®^ subtest 3; and a large effect for UFOV^®^ total. From baseline to posttest 2, small to medium effects were found for UFOV^®^ subtest 3, and UFOV^®^ total; and medium to large effects for NIH Toolbox executive function and UFOV^®^ subtest 4.

Sensitivity analyses (repeated measures ANOVA examining group X time interaction) were generally consistent with the previous ANCOVAs with the following exceptions: a group X time interaction was not found for UFOV^®^ subtest 2 from baseline to posttest 1 or for the UFOV^®^ total from baseline to posttest 2. Furthermore, the group X time interaction for NIH Toolbox episodic memory approached significance (*p* = 0.05).

Finally, while the SOP training group trained an average of 7.40 h (standard deviation = 3.92; range 0–14 h), a post hoc analysis was examined to determine whether adherence to the intervention influenced our models. The prior repeated measures ANOVAs were re-conducted in a subset of participants who were the most adherent [i.e., completed more than 8 h of training (*n* = 17)]. Results were largely consistent with prior analyses in the full sample. We found significant group X time interactions from baseline to posttest 1 for: UFOV^®^ subtest 3 and trends (*p* < 0.10) for UFOV subtest 4, NIH Toolbox working memory, and episodic memory. From baseline to posttest 2, significant group X time interactions were found for NIH Toolbox executive function and UFOV^®^ subtest 4, and trends (*p* < 0.10) for NIH Toolbox SOP.

## Discussion

Cognitive changes are commonly reported among cancer survivors. There is preliminary evidence that computerized cognitive training programs improve cognitive functioning in cancer survivors [11]. However, little is known about the efficacy of this training in the home and in more racially diverse populations. This randomized controlled pilot study evaluated the preliminary efficacy of a home-based SOP intervention, in a racially diverse group of breast cancer survivors living in the Deep South.

The findings were similar to Von Ah et al., in that the SOP training group demonstrated greater improvements in SOP as well as episodic memory compared to the no-contact control group [11]. Findings extend the Von Ah et al. study by demonstrating that these training gains can occur when the SOP training is completed in the participants’ home rather than in a research laboratory setting. In Von Ah’s study, 12% of the participants were African American, whereas over 50% of SOAR Study participants were African American. In the SOAR Study, the SOP training was well-tolerated and beneficial for African American BCS. Finally, the SOAR Study examined participants over a longer follow-up period (6 months versus 2 months post intervention) and therefore is able to demonstrate SOP training benefits over a longer period of time.

SOP training has been shown to improve not only SOP and episodic memory, but also improve everyday functions such as driving ability [18]. Furthermore, prior studies have demonstrated that SOP-related improvements transfer to clinically significant improvements in other health-related quality of life outcomes [19]. For example, using the ACTIVE Study, Ross and colleagues [18] found that, compared to a no-contact control group and a memory training group that served as an active control, SOP training was effective in protecting driving maintenance and driving frequency and exposure over a 5-year period. For BCS who are aging, such SOP may be protective of their driving ability. In the ACTIVE Study, other benefits of SOP were observed over time such as protection from depression [20] as well as improved self-rated health [21], health-related quality of life [19], and locus of control [21]. Thus, SOP may be considered as an adjuvant treatment for symptom management in BCS. Future studies of SOP with BCS should consider these other effects.

Future research should also consider dosage as an important component of therapeutic benefit. In a meta-analysis of 52 domain-specific cognitive training studies with healthy older adults, Lampit and colleagues [22] observed that a therapeutic dose between 10 and 20 h of training may be ideal. More than 20 h of cognitive training may actually yield less therapeutic gain, possibly due to boredom or fatigue. Yet, as exhibited in the ACTIVE Study, booster sessions of cognitive training tended to yield beneficial gains. A future direction should consider whether 10 vs 20 h of SOP training would produce a better or worse therapeutic benefit to participants [23]; in fact, such an approach is being examined in another clinical population of older adults with HIV [24].

Finally, in the same meta-analysis described above, Lampit and colleagues [22] found that when pooling the effect sizes of all of the 52 cognitive training studies, the SOP training studies yielded the highest effect size (*g* = 0.31) compared to attention training (*g* = 0.024) and others. Again, using the ACTIVE Study, compared to the no-contact control group, Edwards and colleagues [23] found those who received the SOP training had a 33% reduction in dementia over a 10-year period. A longitudinal study may find that such training may be of similar benefit with BCS as they age.

## Study limitations and strengths

All studies have strengths and limitations, and this study is no exception. As for limitations, this study has three. First, although an initial sample size of 30 participants in each group clearly satisfies central limit theorem needed for the statistical analyses performed in this study, the sample size limits the generalizability of the findings. Additionally, all participants were from only one geographic location, which further limits generalizability. Second, a number of statistical analyses were conducted in this study but were not corrected for alpha inflation; however, a pilot study with such a small sample size usually does not correct for alpha inflation because such procedures tend to be too restrictive. Thirdly, although this study did have a no-contact control group, it did not control for computer exposure or staff contact. A previous study showed that a no-contact control group served as a sufficient control group in a study examining SOP in older adults with HIV [25]. In fact, another SOP training study in community-dwelling older adults used both a no-contact control group and a contact control (sham) group as a comparison to the active intervention; the two control groups did not significantly differ from each other and both served as an excellent comparison to the cognitive intervention [25]. Albeit, future studies should support the inclusion of a true contact control (sham) condition that also controls for social contact with study staff and computer exposure.

Likewise, this study has four main strengths. First, this sample has excellent representation of African American women (51.7%) as well as Caucasian women (48.3%). In that regard, this study is one of the few BCS cognitive interventions with a large proportion of minority women. Secondly, significant results were found, despite a small sample size, which highlights the magnitude of the effect size of the treatment. Third, for a pilot study, the added feature of having an immediate posttest and a 6-month follow-up was unique in providing novel data to determine whether the intervention was robust over time. Fourth, the attrition rate for this study was remarkably low (5%) after 6 months, which suggests that that the intervention was well-tolerated.
